# Retraction: Country-level indices in predictive models of helminth infections: Perspectives from Southeast Asia

**DOI:** 10.1371/journal.pntd.0014779

**Published:** 2026-10-12

**Authors:** The PLOS Neglected Tropical Diseases Editors

Following the publication of this article [1], concerns were raised regarding the references. Specifically, References 2, 9-13, and 15 could not be retrieved.

Upon editorial follow-up, the corresponding author requested retraction of the article.

In light of the issue with the references that raises concerns about the integrity of the published article, the *PLOS Neglected Tropical Diseases* Editors retract this article.

NKR and SKR agreed with the retraction. CT either did not respond directly or could not be reached.
